# 

**Published:** 1874-02

**Authors:** 


### BOOK NOTICES.

"*Phonography.*" A journal devoted to the promotion of this useful and profitable art, edited monthly by Elize B. Burns, 33 Park Row, New York.

Lee & Shepard, of Boston, and Lee, Shepard & Dillingham, of New York, are, with their usual activity and enterprise, pushing a large number of new and useful books into public notice. Among these are "*Miss Thistledown,*" by Sophie May, 205 pp., with half a dozen illustrations. "*Money Maker,*" by Oliver Optic, 361 pp. 12mo. Boys and young men who aim for success in the business of life will be much instructed in the story of the book.

"*The Arena and the Throne.*" By L. T. Townsend, D. D., author of "Credo," "Sword and Garment," &c., 264 pp., 12mo, with many appropriate illustrations. "Are all worlds inhabited?" is the 1st chapter. The 2d begins "Every man's life ends in defeat or triumph," to which 75 pages are given. "The Triumph," is the 3d chapter, stating that each man's life is a feat and symbol, going back for proof to Abraham and Job, to which latter many pages of absorbing interest are given, ending with the suggestive and comprehensive truth "To pronounce one sinful because in trouble, gives the lie to Gethsemane." The last chapter discusses Pantheism. Messrs. Lee & Shepard have seldom issued a more valuable book—valuable to every cultivated mind.

*Temperance Dramas, Comedies and Farces.* By George M. Baker. Board binding, 230 pp., 25 cents. Is calculated to do a great deal of good. It is well worthy of being made a school book to serve as a text for Thespian performers.

"*Rhoda Thornton's Girlhood.*" By Mrs. Mary E. Pratt. Illustrated by C. G. Bush. An interesting story well told, beginning at the almshouse and ending with sunshiny days.

"*Home Nook.*" 384 pp., 12mo. By Amanda M. Douglass, author of "Stephen Dane," &c., illustrating in the narration of incidents a fact, which everybody knew before, that life has its ups and downs, and yet no one can read the book without deriving consolation and encouragement, nor without having been made better and wiser, by its perusal.

"*Poetical Dramas,*" for Home and School. By Mary L. Cobb. 13 in number, 189 pp., 12mo. "Lady of the Lake," "Spanish Gipsy," "Moses in the Bullrushes," "Queen Vashtie, Deborah and Barek," &c. Also, "Lectures to Young Men and Women," by the Rev. Thomas L. Clark, D. D., L.L. D., Bishop of the Diocese of Rhode Island. 167 pp., 12mo. An unmistakably valuable book, well worthy of being in the possession of every young man and woman. Subjects: "Formation of Character," "True Principles of Trade," "Amusements," "Books," "Purity a Source of Strength," "True Style of Man," &c., &c.

Warming Houses by furnace heat is ruinous to furniture and impairs the health of multitudes. The open fire place, the old fashioned fire on the hearth, is the most unexceptionable of all known methods for keeping our dwellings comfortably warm. The *Low Down Grate* of Thos. Dixon & Sons, of Philadelphia, burns hard coal on the hearth, kindles it without a "blower," compels abundant ventilation, and gives out a cheerful heat.

"*Dental Cosmos.*" $2.50 a year. Edited by JAMES W. W. WHITE, M. D., D. D. S. Although this monthly well merits the patronage of the Dental Profession, every number contains practical information about the teeth, the proper care of them, their nature and structure, of great value; and where there is a growing family of children a more profitable investment for reading matter could scarcely be made, for there cannot be good teeth without good health, and they are essential to good looks everywhere.

"*Scientific American.*" Weekly. $3 a year. Now in its 30th year. Is one of the best conducted newspapers in the country, and in its line it has not an equal in ability in the world. It is not only adapted to the wants of mechanics, inventors and scholars, but as a family paper giving most valuable information of a domestic character and about home life, it merits very general patronage, moral, reliable and self-respecting. Published by MUNN & Co., 37 Park Row, New York.

"*Herald and Presbyter.*" $2.50 a year. 178 Elm street, Cincinnati, Ohio. Has been for thirty-four years a sturdy defender of the Presbyterian Faith, and on that account alone it merits the patronage of that large and influential body of people scattered over the Mississippi Valley and over the plains and forests of the great Northwest. It gives weekly a large amount of current news—general, political, financial and religious.

"*The Christian Intelligencer.*" New York. The organ of the Dutch Reformed Church. $3 a year. During a long course of years has won the respect of all denominations from the dignity, ability and consistency with which it has been managed. Every number contains a large amount of instructive, religious and general reading.

"*The Christian Weekly,*" with many beautiful illustrations, is published at 150 Nassau street, New York, at three dollars a year. The reading matter is adapted to all classes and conditions, and is well calculated to instruct, to elevate and to cherish a healthy, moral and religious sentiment in every household where it is taken. No one can read it without being made better hereby, and it is hoped that it receives, as well as merits, a liberal patronage.

ROBERTS BROTHERS have done an important service to the public in issuing in handsome style, large print, tinted paper, an 8vo. of 377 pages, with portrait, "*The Personal Recollections, from Early Life to Old Age, of Mrs. Somerville,*" with selections from her correspondence by her daughter, Martha Somerville. Mrs. S. was the most learned woman of her century. She published "The Mechanism of the Heavens" in 1831, "Molecular and Microscopic Science" in 1869, "Physical Geography" 1870. Mrs. S. died in 1872. A month longer and she would have entered her ninety-third year. She died in her sleep, as a babe falls away on its mother's bosom, without previous sickness, in the perfect possession of all her mental faculties. A few days before she died she wrote: "Now that I am in my ninety-second year, I must soon expect the signal for sailing. It is a solemn voyage, but it does not disturb my tranquillity. Deeply sensible of my utter unworthiness, and profoundly grateful for the numerous blessings I have received, I trust in the infinite mercy of my Almighty Creator. I have every reason to be thankful that my intellect is still unimpaired, and the infirmities of age are so light to me that I am perfectly happy." In a few days after writing thus she died, showing by herself that persons may live happily, healthfully and enjoyably to near a hundred years. An additional value in the book consists in descriptions of interviews with many of the distinguished men and women who lived during the latter half of the previous century. There is one blemish at the end of the book, which antagonizes much of the pleasure of its perusal, and it may not be too harsh to call it an impertinence on the part of the daughter who edits the volume. She says that her mother "dared early to think for herself in religious matters, and fearlessly shook off those doctrines of her early creed which she thought incompatible with truth, holding quietly but resolutely to the ultimate truths of religion, regardless alike of the censure of bigots or the smiles of sceptics." We all know what that amounts to—a forsaking of the religion of her fathers, looking upon it with a species of contempt—thus living and dying in a faith of her own devising. The book ought to end on page 374, with the last written words of the distinguished autobiography, quoted above.

"*Memories*," published by HARPERS, 339 pages, 12mo, dedicated to Governor Dix, the enlightened statesman, the devoted patriot, the gallant soldier, the elegant scholar, the Christian gentleman and honest man. The full title is "Memories of many men and of some women; being recollections of emperors, kings, queens, princes, presidents, statesmen, authors and artists, at home and abroad, during the last thirty years." By M. B. FIELD.

This is one of the most interesting books of travel, observation and criticism which has appeared for many a day. It can be taken up at any time, opened at random, and read with pleasure and profit. It is a good book for shipboard, for the railway, for a rainy day or a country inn, for the seaside and the spa. Those who have travelled abroad will be impressed with its truthfulness. It is one of those books which can be read over and over again, and with a new interest on each successive perusal, and no doubt will command a large sale.

"*The Ways of Women.*" Published by JOHN P. JEWETT & Co., No. 5 Dey street, New York.

The author is J. V. SMITH, M. D., formerly Mayor of Boston, and for sixteen years the able editor of the Boston *Medical and Surgical Journal*, author of a Class Book of Anatomy, Notes to Cooper's Surgery, etc., etc., and at present President and Professor of Descriptive and Surgical Anatomy in the New York Free Medical College. The book was first published anonymously, but the interest excited in it was so great, and the desire to know the name of the gifted author was so very general, that in this beautiful and enlarged edition the author's name is given as above. The object is to explain familiarly how women may improve their condition by conforming to the laws of health, and how they may be now best qualified for sustaining themselves honorably and successfully in various new relations to society. There are 38 chapters, occupying 491 octavo pages, tinted paper, cloth cover—in short, it is in a style commensurate with the very general as well as high importance of the subjects, some of which are, woman's influence, magnetism, intuitions, power in every age and country, generalization, laws of adaptation, dress, exercise, nervous system, mode of living, etc., etc. Every chapter is a rich mine of information, and every mother of growing daughters, and every young woman of culture, would revel in the reading of this impressive and most practical volume. Three dollars will secure its remission by mail.

